# AWARENESS OF PARTNER PREFERENCES FOR END-OF-LIFE ASPECTS AND TREATMENTS IN OLDER ADULT COUPLES

**DOI:** 10.1093/geroni/igac059.2522

**Published:** 2022-12-20

**Authors:** Sarah Vilpert, Clément Meier, Ralf J Jox, Gian Domenico Borasio, Jürgen Maurer

**Affiliations:** University of Lausanne, Lausanne, Vaud, Switzerland; University of Lausanne, Lausanne, Vaud, Switzerland; Lausanne University Hospital and University of Lausanne, Lausanne, Vaud, Switzerland, 3, Lausanne University Hospital and University of Lausanne, Lausanne, Vaud, Switzerland; Lausanne University Hospital and University of Lausanne, Lausanne, Vaud, Switzerland, 3, Lausanne University Hospital and University of Lausanne, Lausanne, Vaud, Switzerland; University of Lausanne, Lausanne, Vaud, Switzerland

## Abstract

Surrogate medical decision-making at the end of life is common and the patient’s partner is often the person who must make these critical decisions. The challenge of surrogate medical decision-making is to make decisions that best fit the patient's wishes. This study investigates how accurately older adult couples assess each other’s preferences for eleven end-of-life aspects and three medical treatments in a nationally representative sample of adults aged 58 and over living in Switzerland (N=573). The contribution of factors including end-of-life (EOL) discussion and care planning, self-rated knowledge of partner preferences, and EOL preferences homogamy in scoring higher in accurately assessing partner’s preferences for the end of life is examined. Weighted proportions, assessment accuracy scores and ordinary least squares regressions controlled for individuals’ characteristics are calculated. Results show that 44% of respondents accurately assessed their partners’ preferences for aspects related to medical, psychosocial, anticipatory and burden concerns at end of life, and 62% correctly predicted the preferences for cardiopulmonary resuscitation, life-prolonging measures, and palliative sedation. Respondents whose partner discussed their EOL preferences with them, completed an advance directive, designated them as their healthcare proxy, as well as respondents reporting very/rather good knowledge of their partners’ EOL preferences were more likely to have a higher accuracy score for their partners’ EOL preferences. Being a couple is not enough to assess one’s partner’s EOL preferences correctly. Communication about EOL wishes among the couple and individual EOL care planning should be encouraged, as both improve the assessment accuracy of partners’ EOL preferences.

